# The role of arts on prescription in supporting young adults’ mental health and life transitions

**DOI:** 10.1016/j.isci.2026.115463

**Published:** 2026-03-24

**Authors:** Ida Flagstad Hejlesen, Anita Jensen

**Affiliations:** 1 Department of Communication and Psychology, Aalborg University, Aalborg, Denmark; 2 Center for Primary Health Care Research, Department of Clinical Sciences Malmö, Lund University, Malmö, Sweden; 3 University Clinic Primary Care, Skåne University Hospital, Region Skåne, Malmö, Sweden

**Keywords:** health sciences, psychiatry, social sciences, education, psychology

## Abstract

This study investigates the impact of a 10-week Arts on Prescription (AoP) program in Denmark designed for young adults aged 18–30 who were disengaged from education and unemployed. Eighteen participants took part in four semi-structured focus group interviews, and four completed a survey; most reported experiences of anxiety, depression, and stress, with some describing chronic pain, ADHD, or autism. Thematic analysis identified three interrelated themes: catalyst for change, social aspects, and program content and structure. Participants described enhanced confidence, improved mood, strengthened daily routines, and greater readiness to pursue education or employment. Social engagement reduced isolation and supported identity development. At the same time, challenges were noted regarding organizational consistency, perceived safety in certain activities, limited time for relationship-building, and gaps in referral and follow-up. The findings indicate that AoP programs can support wellbeing and transitional processes when accompanied by careful design, safeguarding, system integration, and sustained support.

## Highlights

• Arts on Prescription improved wellbeing, confidence, and daily routines in young adults
• Peer connection reduced isolation and supported identity beyond illness
• Some activities caused stress, fatigue, or discomfort for vulnerable participants
• Referral barriers and limited follow-up constrained program impact

## Graphical abstract

**Figure:**
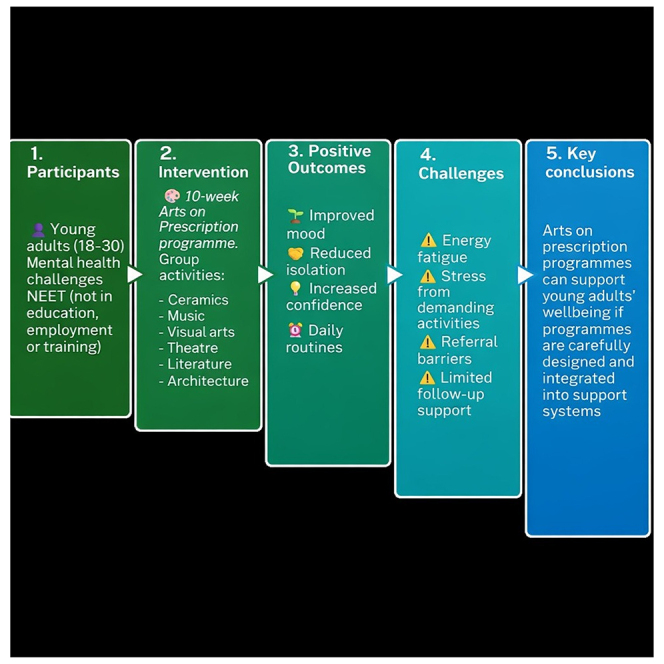

## Introduction

Young adulthood, typically defined as the period between 18 and 30 years of age, represents a critical life stage marked by significant transitions in education, employment, and identity formation.^1^ It is also a time when mental health challenges frequently emerge or intensify, with anxiety, depression, and stress being particularly prevalent.^2^^,^^3^ For many young adults, these difficulties are compounded by coexisting conditions such as chronic pain or neurodiversity (attention-deficit/hyperactivity disorder [ADHD] or autism spectrum disorder), which can further disrupt educational attainment and social functioning.^4^

Growing evidence from multiple countries indicates a troubling rise in mental health disorders among young people in recent years. In Denmark, a study revealed a 36% increase in the number of young individuals aged 12–24 initiating psychiatric medication between 2019 and 2023.^5^ This uptick encompasses treatments for ADHD, depression, and sleep disorders, with a notable rise among young women, particularly those aged 18–24. Similarly, in Norway, the 2023 SHOT study found that 39.7% of female and 25.7% of male college and university students aged 18–35 reported experiencing a current mental disorder.^6^ The most prevalent conditions were major depressive episodes and generalized anxiety disorder Sweden has also reported concerning trends. Approximately 50% of females aged 16–29 have experienced mild or severe anxiety, with 23% reporting severe symptoms.^7^

These figures represent a significant increase from 9% in 2011. Young men also saw an increase, rising from 5% in 2011 to 9% in 2021. Another study, from Australia found that 45.6% of teenagers were affected by chronic or developmental health conditions, including ADHD and autism, which were linked to poor lifestyle factors and mental health issues.^8^ This highlights the complex interplay between mental health and other health conditions in young adults.

In response to the growing need for accessible and holistic mental health support, social prescribing (SP) particularly Arts on Prescription (AoP) programs, has gained increasing recognition as a non-clinical intervention.^9^ AoP involves referring individuals to engage in structured, creative activities as a means of promoting wellbeing and social connection.^10^

Evidence suggests that participating in such arts-based interventions can lead to improvements in self-esteem and motivation, and overall mental health wellbeing, especially among individuals experiencing social isolation or psychological distress.^9^^,^^11^^,^^12^ Furthermore, there is a potential for arts- and culture-based strategies to help meet the current global mental health challenges.^13^

While many arts-based intervention are directed at young people who are challenged with mental health issues and have shown positive health and wellbeing outcomes, they tend to be delivered within the creative art therapy sphere^14^ and scholars have noted that existing literature on young people, art therapy, and health is both limited and highly heterogeneous.^15^

This study explores the impact on young adults who participated in an AoP program (Culture Vitamins for Young Adults). By directing attention to this often-overlooked demographic, the study seeks to examine the role of AoP in promoting mental health and wellbeing among young adults who have disengaged from further education and are not currently participating in the labor market. This article builds on findings from a previous publication.^16^

## Results

The qualitative findings are presented as three main themes: (1) catalyst for change; (2) social aspects; and (3) program content and structure. These themes illustrate how young adults experience participation in the Culture Vitamins program and its perceived impact on their psychosocial wellbeing (see Table 1).

**Table 1:** Main themes, sub-themes, and selected quotations

Main theme	Sub-theme	Quotations
Catalyst for change Description: refers to the ways in which participation in the program initiated positive personal shifts including improvements but also taxing challenges	Mental health improvement	“*My depression has decreased. [It helps] being together with people who have the same or something similar*” (Karla)
	Isolation reduction	“*When you have been on sick leave, just getting out and interacting with other people does you good*” (Frida)
	Routines	“*It’s nice to have something to get up for, and then you get a sense of the weekend again.*” (Aksel)
	Challenges	“*You feel you need some days to recover [from the activity]* (Olivia)
Social aspects Description: captures participants’ experiences of relational connection, both supportive and disruptive—on engagement and attendance	Shared experiences	“*It’s nice to finally feel that you’re on the same wavelength with someone.*” (Clara)
	Safe and caring environment	“*You can just show up, and others know that if you’re having a hard time, then that’s how it is*” (Luna)
	Group dynamic challenges	“*I felt that when people dropped out, it also became easier for me to report sick*” (Aksel)
Programme content and structure Description: how creative activities, facilitation, and organizational design of the program shaped participants’ experiences	Creative participation	“ *I didn’t think I was particularly creative at all, but I just thought it was super cool.*” (Frida)
	Facilitators	“*It’s almost hard to show up and not at some point get in a good mood, because he [coordinator] just has that: ‘It’s just cool to be here’-vibe and that rubs off sometimes.*” (Marlene)
	Structural issues	“*He [coordinator] takes us by the hand and then he just lets us go. From one day to the next. Now I’m standing on my own.*” (Frida)

### Catalyst for change

All participants reported that participation in Culture Vitamins for Young Adults had positively impacted their mental health across several dimensions of psychosocial wellbeing. Many described that participation had helped ease depression and boosted their self-confidence. As Amalie reflected: “*I can feel that I’m getting better. I’m getting better in my depression*,” while Marlene noted how “*Culture Vitamins is good at confirming that you can do lots of small things – that you’re not hopeless at everything*.” Participation seemed to hold special value for the participants who had self-doubt, as it reinforced self-confidence by presenting feelings of achievement.

More than half of the participants expressed that being part of the program had helped reduce their feelings of social isolation. The social aspects of the program were specifically valued by participants on long-term sick leave. As Frida explained: “*When you’ve been on sick leave, just getting out and being around others helps you feel better*.” Participants described how the program also gave a timely mental break from personal struggles. Freja noted: “*It makes you forget certain things during those hours you’re away from home*,” while Frida described: “*You get your head focused on something interesting, rather than just dwelling on being ‘on sick leave’*.”

Participants emphasized the importance of the structure and routines that the program provided. Planned activities gave many participants a sense of purpose. As Frida explained: “*Having something to get up for and to get out the door at a specific time*.” This routine helped prepare for work or study in the future, participants explained, for instance, Aksel: “*The thought of having to show up for something regularly has become less scary because of this*.” In this way, the program became a catalyst to engage more with life, including education and work, which many participants reported. Amalie commented that it was “*a digestible transition that has given me some faith in myself and somehow prepared me*,” while William noted: “*It has been a helpful stepping stone to getting out*.”

This transition seemed to be closely linked to participants developing greater self-understanding of their needs, challenges, and strengths. Frida explained: “*I know that at the library I couldn’t concentrate on just sitting and listening*,” while Marlene pointed out: “*I have learned more about myself … It has helped test what has been really energy demanding*.” Several participants had started making concrete plans for school or work. Noah said: “*I have applied for education as a graphic designer*.” Others were still exploring their options, like looking for internships. Marlene spoke optimistically about her future, suggesting she is ready to move toward work or study while being honest about her current needs: “*After the summer holidays, I hope I can get started with something where there aren’t big expectations of me*.”

However, the program also created challenges for a few participants. Program demands led to psychological stress, making participation a source of tension. Olivia described this: “*Because I have stress, it’s been a bit hard to say no to things, because you want to participate … so you also feel a bit guilty about not showing up*.” Feeling guilty about missing sessions could paradoxically increase mental health pressures. Energy levels were another concern, with participants reporting mixed or negative effects. Olivia described her contrasting experience: “*Some days I’ve gotten a lot out of it and gained energy, and other days - the days where we did something demanding - I’ve actually felt a bit bad afterward and had a dip [in energy]*,” while Alma pointed out: “*I think you can get tired quickly. I do at least when I come home*” and others agreeing about being “tired in the head.” These energy struggles suggest that while the program aimed to energise participants, it sometimes had an adverse effect.

### Social aspects

The social dimension emerged as a cornerstone of the participants’ experience. All highlighted the value of connecting with other individuals who understood their situation and shared their experiences. Frida stated: “*It’s nice to finally feel that you’re on the same wavelength with someone*.” Mutual understanding created a space where participants did not need to explain their circumstances. This was described as particularly important because meeting others in similar situations helped normalize their feelings. As Karla put it: “*You don’t get viewed as strange because you’re having a hard time*.”

Participants described the groups as safe spaces built on respect and support. Freja shared her thoughts: “*I’ve noticed that we care for each other in a different way. We apologise. If someone crosses a line or even thinks they might have, they say sorry. It’s so nice to see that kind of care for each other*.” Several participants mentioned they wanted to continue the friendships they had made during the program. Some intended to keep in touch through group activities like choir, while others planned to use social media. As Marlene said: “*Being able to reach each other and follow each other’s lives a bit*.”

The social experiences appeared to support important identity shifts for some participants. They described moving away from viewing themselves through the lens of illness, unemployment, or as education dropouts. Instead, their focus began to shift toward their abilities and goals. Clara expressed this: “*I have been sick for a long time and have felt that my whole identity is being sick. I thought it was liberating that there was room to be vulnerable, but that you didn’t need to talk about being sick … It was important for me that we were told the first day by the coordinators that you participated in the program because you were other things than just sick*.” In this way, participating may have offered a way to reshape how participants thought about themselves, beyond their challenges.

Beyond the positive impacts, participants also pointed out problems with group dynamics that undermined some of the social gains. Some groups struggled with forming social connections at the start of the program, as Sofia recalled: “*It was very much that you just walked alone and sat alone. We maybe didn’t really dare to talk to each other*.” Building group cohesion took time, with some participants feeling left out in the beginning. When participants dropped out or had irregular attendance, it made it harder for others to stay motivated. Noah commented: “*… when people dropped out, it also became easier for me to call in sick*,” highlighting the importance of group stability for maintaining individual engagement.

Several participants emphasized that they would have appreciated more time for socializing. They noted that some sessions felt rushed, leaving little time for connecting with other participants. In addition, some commented that they would have benefited from bonding with others at an earlier stage in the program and they recommended adding teamwork activities, longer breaks, or other opportunities to get to know each other better. Luna put it: “*We are also there to get to know each other. I think it’s frustrating that many of the activities were so busy, as if we had to fill all the time. Sometimes it might just be nice to have a long break*.”

### Program content and structure

Participants had various comments on the program structure and the activities. In general, the program was appreciated especially because of the emphasis on voluntary participation and lack of pressure to produce something specific. As Carl noted: “*There has never been a time when I’ve been told: ‘Can’t you at least try?’*” This approach, without pressure, was different from other activities the participants had been involved in at school or work settings. Participants who had faced pressure elsewhere found the setup to be beneficial. Marlene commented: “*It felt liberating not to be forced to show up*,” while Freja appreciated “*that it doesn’t matter if you don’t come one day.*”

Participants considered the proactive nature of activities crucial for positive engagement. Ellie said: “*I don’t think I would have kept coming if it was just about looking at art*,” while William proclaimed: “*I wouldn’t have bothered showing up for the other [receptive activity]*.” The hands-on activities seemed to be key to maintaining participation. Several participants found themselves either rediscovering creative hobbies or finding new ones, even some they did not expect to like. Frida noted: “*When I was told we were going to sing, I just thought ‘Fuck’. And now I go to choir.*”

The creative sessions provided some experiences of success. Participants described developing various skills progressively, such as clay-shaping techniques, and highlighted the satisfaction of creating products they could be proud of. To reconnect with creativity became a highlight of the program for many. Ellie shared: “*I love being creative, and I love it even more after this*,” while Marlene reflected: “*It brought out how much I enjoy being creative.*”

All participants provided positive feedback about the AoP coordinators. However, the comments about the art guides from the participating arts and culture institutions were more mixed. Participants recalled the guides to be lively and engaged but also shared some undesired experiences. Luna described one guide as “*arrogant*” and observed that some guides “*became irritated if we did not have questions*”—observations that several of the others agreed upon.

About half of the participants pointed out different structural challenges. While the program structure was generally well-received, with William noting: “*I think the way it’s structured is really good for me personally*,” some practical issues stood out. Meeting times and locations were sometimes inconsistent, which caused confusion for some participants. Participants also pointed out issues with the content. For example, that they did not get enough warning about material that could be upsetting. Frida shared her thoughts: “*I think it’s very inappropriate to show a film [with dead bodies] to a group with people who are in a vulnerable state of mind.*”

Apart from inadequate content warnings, some participants noted that certain activities made them uncomfortable, particularly theater workshops that required participants to perform in ways that did not feel safe. Luna described feeling pushed into a situation: “*The worst was that theater thing … I didn’t want to, but because he kept going and going, I said yes, otherwise we wouldn’t move on … afterward I felt like I hadn’t listened to my own boundaries … again.*” This suggests that facilitators might need to pay closer attention to participants’ comfort levels when engaging in exposing activities, particularly given the vulnerable state of some of the participants.

The referral and access process created barriers, with several participants having to push hard to join the program. William reported: “*I had to pressure my jobcentre officer to get permission*,” while Luna added: “*My jobcentre officer had never heard of Culture Vitamins and wouldn’t refer me to it.*” The lack of follow-up support after finishing the program was also a concern. Noah pointed out this issue saying: “*There could be more help going forward.*” Many participants would, after the AoP program, have to move on to the jobcentre’s mandatory activation program, and Noah’s comment suggests this was a considerable step. Additionally, Freja shared her thoughts commenting: “*Now it’s starting to work, and then it’s over.*” She was concerned about ending the program just as it seemed to start helping her, needing more time to reinforce positive patterns.

## Discussion

In our study, we found that the AoP program for young adults with mental health challenges had a positive impact on the psychosocial wellbeing of the participants. Through three themes, we have contextualized the benefits of participation. (1) Catalyst for change: participation acted as a catalyst for personal change by supporting mental health improvement, reducing isolation, fostering daily routines, and presenting challenges that required adjustment. (2) Social aspects: the program created meaningful social connections through shared experiences and a safe, caring environment, while also revealing how group dynamics could influence participants’ motivation and consistency. (3) Program content and structure: engagement was shaped by the creative activities, the supportive and motivating role of facilitators, and structural elements of the program that at times enabled growth but also introduced challenges related to continuity and independence. Here, we discuss our findings.

### Enhancing social connectedness in group-based art activities

Participation in the group-based AoP program functioned as a catalyst for change, with participants describing improvements in mental wellbeing, reduced isolation, and the re-establishment of everyday routines. Engagement in creative practices such as music, visual arts, literature, and drama provided participants with a reason to leave home, structure their week, and reconnect with others, which they perceived as contributing to improved mood and motivation. These experiences align with broader evidence that group-based arts activities can support mental health and psychosocial development in young adults through interrelated emotional, cognitive, and social mechanisms.^17^ In particular, music has been shown to activate reward and motor networks in the brain, supporting emotional integration and embodied wellbeing.^18^

The social aspects of the program emerged as central to participants’ experiences and appeared to underpin many of the reported wellbeing benefits. Peer-to-peer interaction was naturally fostered through shared creative activities, enabling participants to connect with others who had similar lived experiences of mental health challenges and social exclusion. For young adults who are not engaged in education, employment, or training (NEET), opportunities for stable social anchoring are often disrupted, leaving them more vulnerable to social isolation and its negative impact on wellbeing.^19^

This is especially significant given that emerging adulthood is a developmental stage marked by heightened identity exploration and a strong need for belonging.^3^ The findings align with the “Social Cure” model^20^ which emphasizes how group identification and shared social identities can promote resilience, belonging, and wellbeing.

However, participants’ accounts also indicated that social connection did not arise automatically from arts participation alone. Given the high levels of isolation reported prior to program entry, the benefits of engagement depended on the active cultivation of trust, safety, and mutual understanding within the group. Some participants expressed a desire for more time allocated specifically to informal social interaction, suggesting that, for young adults, structured creative activities may need to be complemented by explicit community-building practices. This contrasts with findings from AoP studies involving older adults, where participation in the activity itself often appears sufficient to generate social connection.^10^^,^^21^ Additionally, a small number of participants described discomfort when asked to engage in activities they perceived as unsafe, such as a theater performance, highlighting the importance of clear communication, consent, and adequate preparation when introducing potentially boundary-testing content. These findings raise important ethical considerations regarding arts programs for vulnerable groups, including whether such activities should be accompanied by trigger warnings.^22^

Beyond social connection, participation in creative activities contributed to a growing sense of competence and self-efficacy, reinforcing the program’s role as a catalyst for broader life changes. Several participants described increased confidence in their creative abilities, which in turn appeared to influence their willingness to consider re-engagement with education or employment. This finding is consistent with Bandura’s^23^ theory of self-efficacy, which posits that successful engagement in meaningful activities strengthens perceived control and motivation. Importantly, this transfer of confidence was not immediate or guaranteed; participants’ wellbeing trajectories toward the end of the program were shaped by the level of post-program support they received. Those who experienced supportive and collaborative transition planning reported sustained optimism, whereas participants who felt pressured or unsupported described a decline in wellbeing, highlighting the need for continuity of care beyond the AoP intervention.^12^

### Unique needs and participation barriers for young adults in AoP programs

Our study highlights that the young adults in the AoP programs experienced unique developmental needs and participation barriers that shape their engagement and outcomes. Many participants were in a transitional stage, having discontinued further education due to mental health challenges and remaining disengaged from employment or training—a period that can be described as a “developmental limbo.” In this context, participation in group arts program over 10 weeks offered structured opportunities for experimentation, reflection, and self-construction, allowing participants to reclaim personal narratives and develop empowering identities.^24^ Participants described the arts as central to “becoming someone” and “finding direction” amid emotional turbulence, echoing findings from other community-based arts programs for young people with mental health challenges.^17^

The catalyst for change observed in our study was influenced by the complexity of participants’ needs. Several participants had co-occurring conditions—including neurodiversity such as ADHD or autism, chronic pain, and mental health challenges—necessitating nuanced approaches to program intensity and inclusivity. Participants emphasized the importance of pacing and opportunities for rest following demanding activities, demonstrating that creative engagement must be carefully calibrated to avoid overwhelming participants while still fostering growth and self-efficacy. These findings reflect what^25^ identified in their systematic review as “misalignment producing no response”—their synthesis of 19 studies focusing on SP found that such unbalance resulted in absence of positive engagement or outcomes and frequently led to distress. The findings highlight the importance of matching SP referrals to patients’ willingness and ability to engage.

Group dynamics also played a pivotal role in participation. While the participants valued the “freedom without pressure” inherent in the voluntary nature of the program—supporting autonomy and personal agency—this flexibility sometimes contributed to dropout when peer dynamics were negative. The importance of positive group dynamics is pointed out in other studies.^9^^,^^26^ Implementing safeguards: advance description of session content and triggering elements, training in recognizing trauma-informed boundaries, flexibility within activities to reduce pressure for uniform engagement, and ongoing check-ins that allow facilitators to make adjustments could ensure that challenges are chosen, informed, and calibrated to the needs and capacities of the individual participant—the hallmark of trauma-informed care.^27^ This would regard both the creative and relational benefits of group arts activities such as AoP.

System navigation barriers were also stated, emerging as an obstacle throughout the participants’ journey. Several participants described difficulties when attempting to access the program through existing referral routes, requiring advocacy from family members or persistence with case workers who seemed not to be aware of AoP possibilities. Similar findings have been noted in other studies.^12^^,^^28^

The lack of system consistency appeared to impact negatively on participants’ sense of agency and self, with some stating they felt “not listened to” by their jobcentre officer, and dreading returning to services that had already disempowered them. In addition, the absence of coordinated follow-up support after program completion exacerbates participants’ difficulties in navigating subsequent steps, aligning with findings from other studies that highlight the importance of structured transitions for AoP participants.^29^

Collectively, these findings emphasizes that AoP programs for young adults must integrate flexible, inclusive, and socially supportive structures that address both the developmental vulnerabilities and systemic barriers experienced by this population. By aligning program intensity with participant capacities, fostering safe and meaningful social interactions and providing structured pathways for continuation and follow-up, AoP interventions can more effectively act as catalysts for positive psychosocial change.

This study demonstrates that AoP programs can provide meaningful psychosocial benefits for young adults, supporting mental health, reducing isolation, and fostering identity development through creative engagement and social connection. At the same time, the findings underscore that young adults may have unique developmental needs that require tailored approaches. Challenges such as fluctuating energy levels, feelings of guilt related to attendance, and exposure to activities perceived as unsafe highlight the necessity of balancing autonomy with supportive structure and safeguarding participants’ wellbeing.

Structural barriers, including difficulties navigating referral pathways and the lack of coordinated follow-up, further limited the program’s impact, pointing to the importance of embedding AoP interventions within broader systems of care and support. Importantly, the results suggest that group-based arts activities hold particular promise when they intentionally cultivate social connectedness and self-efficacy, both of which are critical for young adults navigating transitions disrupted by mental health difficulties.

Overall, AoP for young adults can act as a catalyst for change, but its effectiveness depends on sensitive adaptation to developmental context, integration with social and health services, and the provision of sustained opportunities for participation. Future research should further explore how different forms of creative engagement interact with participant wellbeing in this age group, and how structured transitions from AoP to education, employment, or community engagement can be best supported.

### Limitations of the study

This study contributes to the underexplored area of AoP for young adults, a demographic with distinct challenges and opportunities that have not been well represented in previous research. By employing a qualitative approach, the study complements the quantitative orientation of much AoP research, offering deeper insight into how young adults experience the program and how participation intersects with mental health and life transitions.

However, several methodological limitations must be acknowledged. While using a qualitative lens is a strength in that it enables rich, in-depth exploration of participants’ perspectives and contextual nuances, the primary limitation lies in the limited generalizability of findings beyond the specific study context. The sample consisted only of participants who completed the program and volunteered for interviews, introducing potential selection and positivity bias, particularly given known attendance issues. Consequently, the findings may not capture the perspectives of those for whom AoP was less suitable or accessible, echoing challenges identified in previous research.^10^

The study’s single-site design within the Danish welfare system also constrains generalizability, as structural and organizational features may not apply elsewhere. Furthermore, risks of social desirability bias are present, especially in group-based interviews, though these may have been mitigated by conducting interviews within established groups, which participants described as “safe spaces.” This familiarity appeared to reduce anxiety and support openness, while also contributing to reported gains in social confidence. Nonetheless, one-to-one interviews are recommended for future studies to surface divergent perspectives that may not emerge in group contexts.

## Resource availability

### Lead contact

Further information and requests for resources and reagents should be directed to and will be fulfilled by the lead contact (anita.jensen@skane.se).

### Materials availability

There are restrictions to the availability of qualitative data due to ethical and confidentiality considerations, as participants could be identifiable from detailed narratives.

### Data and code availability

• The qualitative data generated and analyzed during the current study consist of focus group interview transcripts. Due to the sensitive and potentially identifiable nature of the data, they are not publicly available. Sharing full transcripts would compromise participant confidentiality and violate the conditions of informed consent and applicable data protection regulations. De-identified excerpts supporting the findings are included in the article. Further information about the study procedures may be obtained from the corresponding author upon reasonable request and subject to institutional and ethical approval.
• This paper does not report original code.
• Any additional information required to reanalyze the data reported in this paper is available from the lead contact upon request.

## Acknowledgments

The authors thank the participants for generously sharing their time, experiences, and reflections as part of this study. Their openness and engagement made this research possible.

## Author contributions

I.F.H. contributed to the conception and development of the research design, and ethical approval process; I.F.H. recruited the participants and conducted the interviews; I.F.H. and A.J. conducted data analysis and data interpretation. Both authors contributed to the writing reviewing and editing the manuscript and approved the final manuscript for submission.

## Declaration of interests

The authors declare no competing interest.

## STAR★Methods

### Key resources table

**Table:** 

REAGENT or RESOURCE	SOURCE	IDENTIFIER
**Software and algorithms**		

SurveyXact	N/A	https://www.ramboll.com/products/energy/surveyxact

**Other**		

Six steps thematic analysis	Braun & Clarke^30^	N/A

### Experimental model and study participant details

#### Participants

The study included 22 participants (female n = 15, male n=5) aged between 18 and 30 years old who had reported mental health challenges including anxiety, depression, or stress. Several participants also had coexisting conditions such as chronic pain, ADHD, or autism. Most participants had completed High School, and several had begun higher education courses but had either interrupted or discontinued for health reasons higher education and were not in the job market (NEET- Not in Education, Employment, or Training), Three of the participants had participated in the AoP programme previously but were referred again as they had missed many sessions the first time (due to adjustments to new medication).While the completion rate was 87% on the overall programme, researcher observations in Spring 2023 revealed frequent low attendance (down to just 1-2 participants per session). Participation was not formally documented, as attendance was entirely voluntary. The groups were closed and only included referred participants.

#### Referral process

Healthcare professionals (GPs) or case workers at the local jobcentre referred participants to the Culture Vitamins for Young Adults programme. Participation was optional, and there were no sanctions for absence (e.g. loss of financial unemployment benefits). Once referred, the AoP coordinator invited each participant to a one-on-one introduction meeting. The AoP coordinator was available at the jobcentre once a week to respond to inquiries from individuals interested in the programme.

#### Inclusion criteria

The study and programme included young adults aged 18-30 years old with mental health challenges who had dropped out of their education or were unemployed. No exclusion criteria were set since the programme focused on including all young adults in the target age group facing mental health challenges that were seen as a barrier for continuing education or being active in the job market that interfered with their ties to work or education.

#### Recruitment

At the end of every 10-week programme cycle in Spring 2023, participants were invited to join group interviews to share their experiences. Participation was voluntary. It was also possible to share experiences by completing an online questionnaire.

#### Ethics

The study was approved by the Scientific Ethics Committee for the North Denmark Region (Journal no: 2022-000764). Participants received both written and verbal information and consented in writing to take part in the study. Data were handled following the rules set by the Danish Data Protection Agency. All participants are anonymized and pseudonymized.

### Method details

#### Programme design

Culture Vitamins for Young Adults lasted 10 weeks with activities approximately three times per week (28 activities in total, including one introductory meeting). Each session ran for 2-3 hours. Groups included a maximum of 15 participants. Professional guides from various arts and cultural institutions led the activities. Table 2 provides details regarding place, activity, and frequency of sessions (see Table 3 for further details of the activities).

**Table 2:** Arts and culture activities included in the AoP program

Arts and cultural institution	Cultural activity	Number of sessions
Ceramics Aalborg	Ceramics	5
North Urban Art Studio	Art workshop	6
Nordkraft (Den Rytmiske)	Group singing	5
Teater Nordkraft/Aalborg Theater	Theater	3
Aalborg Library	Guided shared reading	3
Utzon Center	Architecture workshop	3
Aalborg City	Street art	1
The Cinema Aalborg	Guided film experience	1
Jazz9TUS	Jazz session	1

**Table 3:** Description of AoP program activities

Arts/culture activity	Description	Cultural institution	Facilitator
Ceramics	The ceramics activities comprised five sessions designed to introduce participants to a range of technical and creative methods in clay work. Techniques covered included pinching, coil building, slab construction, hand-building, and the Kurinuki method (hollowing forms). Each session commenced with a structured introduction to a specific technique, followed by independent practice in which participants applied the methods to their own projects. Works were dried between sessions, enabling glazing of previously created pieces alongside the introduction of new techniques. Sessions were approximately 3 h in duration, with flexibility for participants to take breaks as needed.	Ceramics Aalborg	Ceramics instructor
Art workshop	The visual arts activities consisted of six sessions in which participants engaged with a variety of visual art forms, including drawing, painting, and spray art. The sessions emphasized the creative process, encouraging participants to reflect on the meaning of creativity and to develop their own creative capacities. Activities included both individual and collaborative projects, providing opportunities for skill development and peer learning. Sessions were conducted in a dynamic creative environment where independent artists worked in parallel, offering participants indirect exposure to diverse artistic practices.	North Urban Art Studio	Artists
Group singing	The group singing was delivered over five sessions and facilitated by a vocal instructor who also provided piano accompaniment. Participants selected repertoire collaboratively from a catalog of approximately 50 well-known popular songs spanning several decades, supplemented by additional suggestions from the instructor. Each session comprised two structured singing periods of approximately 50 min each, separated by a 15-min break. The format was designed to combine guided musical instruction with participant choice and engagement in collective music-making.	Nordkraft (Den Rytmiske)	Vocal Instructor
Shared reading	The shared reading was conducted over five sessions and facilitated by a librarian at Aalborg Libraries who was also a shared reading guide. Each session involved the guide reading aloud from selected texts, with discussion breaks approximately every 10 min to allow participants to reflect on and collectively explore the content. The chosen materials included short stories and biographical works authored by young writers or addressing themes relevant to young adults. In addition to the reading sessions, participants were introduced to the library’s facilities and services through a guided tour.	Aalborg Library	Librarian/Shared reading guide
Theater	The theater workshops were delivered over three sessions and facilitated by a theater instructor. The workshops introduced participants to the operations of a professional theater through meetings with staff working both on stage and backstage, as well as opportunities to attend rehearsals or current performances when available. Participants engaged in practical theater exercises, including costume selection for roles, script reading, and exploration of different aspects of performance. The sessions were designed to provide both insight into theater production processes and hands-on experience with elements of the creative practice.	Aalborg Teater/Teater Nordkraft	Theater instructor
Architecture workshop	The architecture workshop was held over three sessions at the Utzon Center and combined guided tours with practical exercises. Participants were introduced to the building and to Jørn Utzon’s architectural approach, providing both historical and theoretical context. Practical activities included sketching designs on paper and constructing models using unconventional materials such as LEGO blocks and orange peels. The sessions aimed to integrate architectural theory with hands-on creative practice, enabling participants to explore design processes in an accessible and experimental format.	Utzon Center	Architecture instructor
Mural tour	The guided city walk took place in Aalborg’s city center and was led by the same artist who facilitated the art workshop. Participants were introduced to the city’s numerous wall paintings through a relaxed walking tour, which included scheduled breaks. The tour combined direct engagement with the artworks with narratives about their creation and the artists behind them, thereby situating the works within a broader cultural and artistic context.	Aalborg City	Artist
Guided film experience	The cinema activity consisted of a single session facilitated by a member of the cinema staff and AoP coordinator. Participants attended a screening of a current feature film selected for its thematic relevance to young people, addressing issues such as love or mental health. Following the screening, a 30-min group discussion was conducted by the cultural coordinator using prepared guiding questions to support participant reflection and dialogue on the film’s themes and their personal responses.	The Cinema Aalborg	Cinema staff member
Jazz Session	The jazz activity was conducted as a single-session event at a local concert venue. Participants observed a jazz band during rehearsal prior to their evening performance and received an introduction to the band and to jazz music more broadly from the concert promoter. The session included opportunities for direct interaction with the musicians, as well as an informal meal shared among participants during a break. Participants were also given the option to remain for the evening concert. The venue’s regular hosting of international bands provided an authentic and intimate jazz environment for the activity.	Jazz9TUS in Jomfru Ane Street	Promoter and artist

The programme offered participants different creative experiences focusing on participation and creative expression. An AoP coordinator participated in the activities throughout the programme alongside the participants and the arts facilitators.

#### Data collection

The study used a qualitative research approach, organising four focus group interviews with groups of four, four, eight, and two participants. The interviews were conducted at a place familiar to the participants. While adopting a qualitative lens to prioritise depth and contextual understanding, we acknowledge that the findings are interpretive in nature and not intended for statistical generalisation.

In addition, four other participants gave written answers to the questions using SurveyXact. Each focus group interview lasted between 1 to 1.5 hours and was scheduled during the last activity of a 10-week programme cycle. The interviews were conducted by the first author from February to July 2023, using a semi-structured interview guide (see supplemental file 1). All interviews were in Danish, recorded, and later transcribed.

#### Data analysis

A thematic analysis, as described by Braun and Clarke (2022),^30^ was used to analyse the data. This process unfolded in six steps. The first step involved getting familiar with the data by reading the transcripts. The second step focused on creating initial codes from the data. Thirdly, researchers developed meaningful themes and sub-themes from the coded data. By the fourth step, they reviewed the themes to ensure they connected well with the coded extracts and the entire dataset. In the fifth step, they defined and organised the final themes. Finally, they combined their analysis and data excerpts into a narrative during the writing phase.

Both authors worked separately to code the data and build themes (steps 1-2) then later came together to share their perspectives and agreed on the findings (steps 3-6). Table 1 outlines the main themes, along with their related sub-themes, which illustrate what participants experienced in the Culture Vitamins for Young Adults programme. Three main themes were found, and various sub-themes.

### Quantification and statistical analysis

No statistical methods, sample sizes, and software was used generated in the study.

## Supplemental information

Document S1. Method S1
