# Supplementary material for: The role of arts on prescription in supporting young adults’ mental health and life transitions
Source: iScience. 2026 Mar 24;29(4):115463. doi: 10.1016/j.isci.2026.115463 (PMC13090950; doi:10.1016/j.isci.2026.115463)
Supplement: Document S1. Method S1 [file mmc1.pdf]

**iScience, Volume *29***

## Supplemental information

# The role of arts on prescription in supporting young adults' mental health and life transitions

**Ida Flagstad Hejlesen and Anita Jensen**

**Method S1: Interview Guide for Participants: AoP for Young Adults**

**Overall**

**• Now that you are at the end of Culture Vitamins, what do you think of the programme overall?**
**Activities**
• Which activities did you like the most, and why?
• Which activities did you like the least, and why?

**Social**
• How has it been to experience art and culture in a group?
• How has it been to exchange experiences with others in the same situation as yourselves?
• There has been a relatively high dropout rate among participants. What significance has this had?

**Duration**
• What do you think about the programme's duration of 10 weeks and the number of activities per week?
• What do you think about the length of each activity (two to three hours)?

**Recruitment**
• How did it work being introduced to Culture Vitamins through your advisor?
AoP Coordinator
• How was your experience with the AoP coordinators and your relationship with them?

**Cultural Guides**
• How was your experience with the different cultural guides?
• In your opinion, what characterizes a good cultural guide?

**Physical and Mental Health**
• What has Culture Vitamins meant for your everyday wellbeing?
• What has Culture Vitamins meant for your physical health?
• What has Culture Vitamins meant for your mental health?

**Future and Connection to Education and Work**
• How do you feel about Culture Vitamins ending?
• What thoughts do you have about what will happen next?
• How has Culture Vitamins influenced your thoughts and opportunities for starting or continuing education or work?

**Participation in Art and Culture**
• In Culture Vitamins for Young Adults, the focus has been on activities where you actively participate in art (e.g., making things with your hands) rather than only observing art. What do you think about that?
• How would you describe your participation in art and culture before the programme (could be anything from going to the theatre to playing music, painting, etc.)?
• Has Culture Vitamins influenced your participation in art and culture?

**Changes**
• Is there anything about Culture Vitamins you wish had been different or could be improved?
